# Depression trends in Hepatitis-C PCR positive and PCR negative patients

**DOI:** 10.12669/pjms.38.1.4788

**Published:** 2022

**Authors:** Qutabuddin Khuhro, Hafeezullah Shaikh, Washdev, Shahkamal Hashmi

**Affiliations:** 1 Qutabuddin Khuhro, National Institute of Gastrointestinal Disease, Dow University Hospital, DUHS, Ojha Campus, Karachi, Pakistan; 2 Hafeezullah Shaikh, National Institute of Gastrointestinal Disease, Dow University Hospital, DUHS, Ojha Campus, Karachi, Pakistan; 3 Washdev, Institute of Behavioral Sciences, DUHS, Karachi, Pakistan; 4 Shahkamal Hashmi, School of Public Health, DUHS, Karachi, Pakistan

**Keywords:** Depression, Hepatitis-C, Interferon, DAA

## Abstract

**Objective:**

To determine the frequency of depression in Hepatitis-C patients and its association clearance of HCV

**Methods:**

It is cross sectional study that was conducted between 1^st^ July to 31^st^ December, 2020, at National Institute of Liver and Gastrointestinal Diseases (NILGID),, Dow University of Health Sciences (DUHS).. Both male and female patients aged 18 to 60 years presenting with Hepatitis-C PCR positive or had received DAA for three months and became PCR negative were included in this cross sectional study. Depression was analyzed by Hamilton Depression Rating Scale (HDRS). Mean and standard deviations were calculated and analyzed.

**Results:**

Total 210 patients were included in this study, with mean age 36.06 ± 10.11 years. Depression was present in 118 (56.2%) patients. Among patients with HCV PCR positive depression present in 63 (30.0%) patients while in HCV PCR negative 55 (26.0%) patients. Similarly, depression in HCV PCR positive male patients, aged ≤40 years 80 (38.1%) and in HCV PCR negative 56 (26.7%) patients.

**Conclusion:**

Patients with chronic Hepatitis-C commonly suffer from depression. However, our study found no significant difference with change in PCR status at 12 weeks.

## INTRODUCTION

Hepatitis-C is the commonest cause of chronic liver disease is CHC (Chronic Hepatitis-C) in the current era.^1^ Hepatitis-C virus (HCV) infection has wide spectrum of hepatic and extrahepatic symptoms including neuropsychiatric condition of depression.^2^ The depression is a clinical disorder with high prevalence among Hepatitis-C population as compared to general population, hence it increase challenges and doubles the burden of disease.^3^,^4^ Frequency of depression in Hepatitis-C infected people in Pakistan is 40% to 60%.^5^,^6^

HCV has role by direct and indirectly in the occurrence of depression.^2^ The mechanism through which HCV effect brain is not fully elucidated yet, it is noted microstructural changes and cerebral metabolite abnormalities consequently results in development of depression.^7^ The symptoms of HCV associated depression can be described by intracerebral neurobiological mechanism, that can be explained by inflammation of the brain by cytokines, and due to viral particles those cross blood brain barrier.^8^ Furthermore, cured Hepatitis-C patients experiencing substantial symptom improvements in baseline mental health issues, fatigue and trends of depression.^9^

This study focused on impact of DAA treatment on depression and HCV clearance in chronic Hepatitis-C population can be associated with changes in trends of depression. Local data of depression in Hepatitis-C population after advent of DAA in not well documented. Therefore, this study was designed and conducted in local population presenting in a tertiary care hospital. This can be helpful in determination of current pattern of depression and anticipation management of mood disorder in patients.

## METHODS

A cross sectional study was undertaken at National Institute of Liver and Gastrointestinal Diseases (NILGID), Dow University of Health Sciences (DUHS) for duration of 1^st^ July 2020 till 31^st^ December 2020, having sample size of 210 patients in two groups of 105 patient HCV PCR positive and 105 HCV PCR negative after Institutional Review Board (IRB) approval from DUHS (IRB-1590 dated 27^th^ June, 2020). Non probability consecutive sampling was used. Both male and female patients aged 18 to 60 years presenting with Hepatitis-C PCR positive or had received DAA for three months and became PCR negative were included. Patients already taking anti-depressants or drugs predisposing to depression such as steroids, or interferon were excluded. Patients were also excluded if they had HBsAg positive, hepatocellular carcinoma, child pugh class B or C, psyshosis and alcoholic liver disease.

Depression was analyzed by Hamilton Depression Rating Scale (HDRS). There were 17 questions in used questionnaire for study with total score ranged from 0 to 54. HDRS scores categorized depression into four categories of severity (score zero to six no depression; score seven to seventeen mild depressions, score eighteen to twenty-four into moderate depression and score twenty five to fifty four into severe depression).

A written consent was taken as per protocol. Patients fulfilling the inclusion criteria were enrolled in the study from (NILGID), Dow University Hospital, Karachi. Demographic data such as age, gender, duration of Hepatitis-C, and status of HCV PCR was noted.

Data was evaluated using Statistical Package for Social Sciences (SPSS) version 22.0. Data was analyzed as percentages and frequencies. Mean and standard deviation were calculated for age and depression score. Frequency and percentage were calculated for gender and severity of depression. Effect modifiers such as age and gender were controlled through stratification. Post stratification, chi square test was applied and p-value of <0.05 was taken as significant.

## RESULTS

Total 210 patients were included in this study. Mean age of the patients was 36.06 ± 10.11 years. Total 136 (64.8%) patients were ≤40 years of age and 74 (35.2%) were >40 years age. Most of the patients had hepatitis duration between 1 to 2 years. Mean HRDS score was 13.30 ± 11.00. Baseline characteristics are summarized in Table-I.

**Table I:** Baseline characteristics of the patients.

	N	%
Age, years	36.06 ± 10.11^*^	
≤40	136	64.8
>40	74	35.2
***HRDS score***	13.30 ± 11.00^*^	
Gender		
Males	112	53.3
Females	98	46.7
***Duration of Hepatitis-C***		
<1 year	34	16.2
1-2 years	89	42.2
2-3 years	61	29.0
3-4 years	19	9.0
4-5 years	7	3.3

* Mean±SD.

Out of 210 patients, depression was present in 118 (56.2%) patients. Total 92 (43.8%) had no depression, 53 (25.2%) had some depression, 33 (15.7%) had moderate depression and 32 (15.2%) had severe depression (Table-II). Among patients with Hepatitis-C, depression was not significantly higher in male patients (Table-III).

**Table II:** Severity of Depression.

Severity of Depression	Percentage
0 to 6 No Depression	43.81%
7 to 17 Some Depression	25.24%
18 to 23 Moderate Depression	15.71%
24 to 54 Severe Depression	15.24%

**Table III:** Comparison of depression with age, gender and HCV PCR status.

	Depression		Total	P value

	Present	Absent		
***Gender***				
Male	68 (32.4%)	44 (21.0%)	112 (53.7%)	0.158^*^
Female	50 (23.8%)	48 (22.9%)	98 (46.7%)	
***Age***				
≤40 years	80 (38.1%)	56 (26.7%)	136 (64.8%)	0.297^*^
>40 years	38 (18.1%)	36 (17.1%)	74 (35.2%)	
***HCV PCR status***				
Positive	63 (30.0%)	42 (20.0%)	105 (50.0%)	0.266^*^
Negative	55 (26.2%)	50 (23.8%)	105 (50.0%)	

* Chi-square test applied.

**Fig.1:**
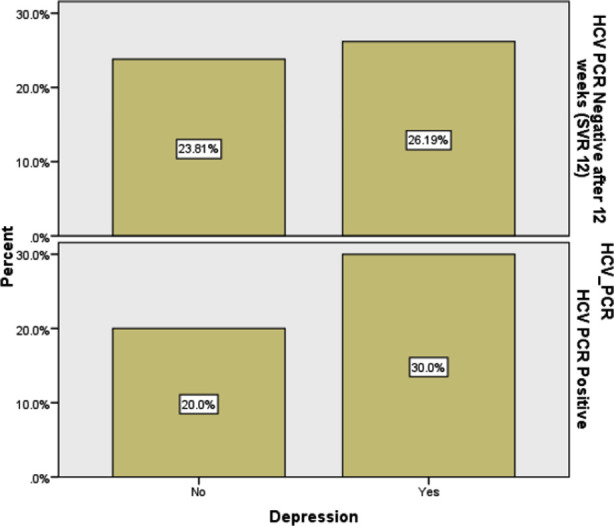
Depression frequency in HCV PCR positive and PCR negative.

Among patients with HCV PCR positive patient’s depression in 63 (30.0%) while in HCV PCR negative 55 (26.0%) (p value of 0.266) patients (Fig.1). Similarly, depression in HCV PCR positive male patients, aged ≤40 years 80 (38.1%) and 56 (26.7%) and in patients who were HCV PCR negative (p value of 0.297). The p-value depicted that no difference in depression among HCV PCR positive and negative patients (Table-III).

The severity of depression based on gender, age group ≤40 years, and HCV PCR status was also not statistically significantly different. (Table-IV).

**Table IV:** Comparison of severity of depression control with patient characteristics.

	Depression severity				Total	P value

	No depression	Some depression	Moderate depression	Severe depression		
***Gender***						
Male	44 (21.0%)	32 (15.2%)	17 (8.1%)	19 (9.0%)	112 (53.3%)	0.442^*^
Female	48 (22.3%)	21 (10.0%)	16 (7.6%)	13 (6.2%)	98 (46.7%)	
***Age***						
≤40 years	56 (26.7%)	41 (19.5%)	19 (9.0%)	20 (9.5%)	136 (64.8%)	0.164^*^
>40 years	36 (17.1%)	12 (5.7%)	14 (6.7%)	12 (5.7%)	74 (35.2%)	
***HCV PCR status***						
Negative	50 (23.8%)	21 (10.0%)	18 (8.6%)	16 (7.6%)	105 (50.0%)	0.354^*^
Positive	42 (20.0%)	32 (15.2%)	15 (7.1%)	16 (7.6%)	105 (50.0%)	

* Chi-square test applied.

## DISCUSSION

Diagnosis with HCV is stressful and affected daily quality of life HCV patinets.^10^ Advent of DAA in 2014 has increased the treatment response HCV patients.^11^,^12^ Thus, knowledge and understanding of ongoing problems of depression can guide healthcare interventions.^13^ Depression have been reported with high frequency in patients with CHC infection resulting impairment to perform daily routine.^14^ Furthermore, contributing factors were being decline in sense of wellbeing, fatigue and knowledge of infection.^15^

The results of our study demonstrate 56.2% frequency of depression in patients with chronic Hepatitis-C. The reported frequency in our study was slightly lower than the one reported in previous local study.^3^ The possible reason could be attributed to the difference in questionnaire used. We used Hamilton Depression Rating Scale (HDRS) to evaluate depression in our study population whereas the previous study utilized Hospital Anxiety and Depression Scale (HADS) for depression analysis.^3^

Another study by Gallegos-Orozco JF et al. reported depression 58.6% slightly higher frequency of depression in patients with chronic Hepatitis-C infection.^16^ A difference in frequency could be attributed to difference that study included all the Child Pugh classes whereas in our study, only patients comprising of Child Pugh class A were included. Since quality of life in patients with Child Pugh class B and C is lower, therefore, this may be a possible reason for increase in depression.

In another study by Schaefer M et al depression was reported up to 70% in HCV patients in the interferon era related to interferon may be due to alteration in monoamine metabolism, high apoptosis rate, altered function of hypothalamic pituitary axis and reduced level of brain derived neurotropic factor (BDNF).^17^ However, that higher rate could be due to a difference in the treatment medication.

The acceptability of DAA in depression is well documented while no conclusive changes in mood disorders.^18^ According to the result of our study, presence of depression was lower in HCV PCR negative (26.2%) patients as compared to PCR positive (30%) patients, however this difference was not statistically significant. However, in contrast to our study Goni-Esarte S et al. found lower frequency of depression which was statistically significant difference of depression score between HCV PCR positive and HCV PCR negative patients.^19^ Furthermore, clearance of HCV virus was associated with improvement of quality of life. It can be explained by one-year long term follow-up same HCV group of patients. 19

Our study results have demonstrated a higher frequency of depression in males with Hepatitis-C as compared to females. In contrast to our study, a previous study by Qureshi MO et al has reported a high frequency of depression in females. 3 This difference could be small sample size of previous study. However, in contrast to our study Song GJ et al. reported female gender was independent risk factor of depression. 20

### Limitations of the study:

It was a cross-sectional study. We did not follow the same population before and after DAA treatment. But to some extent, this limitation has been overcome by taking a same sample size of groups of patients before and after treatment. Another limitation of our study was that health related quality of life (HRQoL) was not evaluated. Third limitation of our study was that we did not include the patients with advanced Child Pugh class (class B and C) patients or patients with hepatocellular carcinoma (HCC). We believe that advanced Child Pugh class and HCC may further increase the frequency of depression in patients suffering from chronic Hepatitis-C infection.

Despite these limitations, we believe that this study was an attempt to evaluate depression among patients treated and untreated for chronic Hepatitis-C in a population of developing country. To the best of knowledge, this is the first of its kind study evaluating depression in chronic Hepatitis-C patients treated with DAA in the country population. It is recommended that a cohort study should be carried out by following a same population so that more insight can be gained on the present topic and effective measures can be taken in management of such patients.

## CONCLUSION

Patients with chronic Hepatitis-C commonly suffer from depression. However, our study found no significant difference with change in PCR status at 12 weeks

### Authors’ Contributions:

**QK:** Conceptualization, Research data collection, manuscript writing, integrity of study.

**HS:** Supervision, Research data collection, evaluation, manuscript writing.

**W:** Research data collection, evaluation of data, review.

**SH:** Methodology, Research data collection, data evaluation, review.
